# Ba/F3 transformation assays

**DOI:** 10.18632/oncotarget.17828

**Published:** 2017-05-11

**Authors:** Kathleen Kong, Patrick Kwok-Shing Ng, Kenneth L. Scott

**Affiliations:** Department of Molecular and Human Genetics, Baylor College of Medicine, Houston, TX, USA

**Keywords:** Ba/F3, cancer, acquired mutation, reproducibility

In the age of large-scale genome sequencing efforts and precision medicine, there is a greater need for rapid functional characterization of newly identified cancer-associated variants with unknown significance (VUS). Since they were first isolated in 1985 [1], the IL-3-dependent Ba/F3 murine hematopoietic cell line has become an increasingly popular system to screen for *bona fide* oncogenic variants found across a wide variety of cancers. In contrast to typical fibroblast or epithelial transformation assays that are relatively time-consuming and less sensitive, the Ba/F3 system benefits from a rapid cellular proliferation rate and the ease of transgene expression via electroporation or retro/lentiviral transduction [2]. The Ba/F3 transformation assay relies on the principle that normal Ba/F3 cells die shortly after withdrawal of exogenous IL-3. Ectopic expression of an oncogenic or “driver” variant in these cells renders Ba/F3 cell survival and proliferation independent of IL-3 and instead reliant on the expressed driver, a phenomenon termed “transfer of oncogene addiction”.

In addition to screening transgenes for transforming potential, the Ba/F3 system can also be used to investigate downstream oncogenic signaling pathways and the susceptibility of driver variants to therapeutics [2]. Driver-addicted Ba/F3 cells die when the driver-engaged signaling pathway(s) is inhibited, and this effect can be rescued by re-introduction of exogenous IL-3 thus providing a counterscreen for off-target effects [2–3]. Such drug studies can determine the potency of inhibitors against wild-type or mutant kinases as well as inhibitor-resistant alleles, predict and confirm inhibitor-target binding modes, and reveal new therapeutic liabilities for oncogenic variants [2]. The Ba/F3 system has been successfully used to screen numerous VUSs and to assess the drug susceptibility of many clinically relevant drivers, including *BCR-ABL* and variants of *EGFR*, *HER2*, *PIK3CA*, and *PIK3R1* [2–4] among others.

In their latest work, Watanabe-Smith and colleagues investigated acquired mutations that arise upon introduction of transgenes into Ba/F3 during standard transformation assays [5]. Despite the increasingly common use of Ba/F3 transformation assays, sequence validation of expressed transgenes in this system is rarely performed. The authors demonstrated that IL-3 withdrawal from oncogenic variant-expressing Ba/F3 cell lines can select for undesired transgene mutations, potentially confounding downstream analyses of the transformed cell lines. Briefly, the authors examined four mutations in three known oncogenic cytokine receptors (*CSF2RB*, *CSF3R*, and *IL7R*) for their studies. By Sanger sequencing transgene-specific PCR products from genomic DNA harvested from retrovirally-transduced Ba/F3 lines before and after IL-3 withdrawal, they determined that the majority of transformed cell lines (those that exhibited IL-3-independent growth) acquired additional mutations in the expressed transgene.

In a series of mechanistic studies, the authors found that (1) the acquired transgene mutations likely result from retrovirus processing and thus exist after transduction but prior to IL-3 withdrawal (2) acquired mutations most often occur in weak oncogenes that require longer culturing in the absence of IL-3 to exhibit their growth-promoting activity in Ba/F3 rather than in strongly transforming oncogenic variants, and (3) time in culture before IL-3 withdrawal did not change the transformation rate of variant-expressing Ba/F3 lines. Although the mechanism of acquired mutation formation is unknown, the authors propose that the inherently mutagenic process of retroviral reverse transcription leads to mutant transgene alleles that integrate into the Ba/F3 genome following transduction. In the case of strongly transforming variants (*CSF3R*^T618I^ or positive control, *BCR-ABL*), selection for the strong drivers would prevent the acquired mutations from reaching the mutation-detection threshold afforded by Sanger sequencing. However, almost all of the cell lines expressing weakly transforming drivers (defined as <1 in 200 cells that exhibit IL-3-independent growth) present with acquired mutations following Sanger sequencing. These mutations can either be functionally active driver variants that proliferate to a level detectable by sequencing, or functionally inert passenger mutations that, by chance, expand alongside the transforming variant. This study emphasizes the need to be cautious of interpreting results using the Ba/F3 system and to have alternative cell lines or orthogonal assays to screen and validate VUSs. Future studies to determine when and how acquired transgene mutations arise in Ba/F3 as well as functionally characterizing acquired mutations will further enhance our understanding and provide hints to counteract this phenomenon. Overall, this work provides useful new information on a popular transformation assay, clearly demonstrating the importance of sequence validating transgenes following Ba/F3 transformation assays prior to initiating downstream biological analyses.
